# Calorimetry Minisensor for the Localised Measurement of Surface Heat Dissipated from the Human Body

**DOI:** 10.3390/s16111864

**Published:** 2016-11-06

**Authors:** Fabiola Socorro, Pedro Jesús Rodríguez de Rivera, Manuel Rodríguez de Rivera

**Affiliations:** Departamento de Física, Universidad de Las Palmas de Gran Canaria, Las Palmas de Gran Canaria E-35017, Spain; fabiola.socorro@ulpgc.es (F.S.); pedrojrdrs@gmail.com (P.J.R.d.R.)

**Keywords:** direct calorimetry, heat conduction calorimeters, isothermal calorimeters, medical calorimetry, non-differential calorimeters

## Abstract

We have developed a calorimetry sensor that can perform a local measurement of the surface heat dissipated from the human body. The operating principle is based on the law of conductive heat transfer: heat dissipated by the human body passes across a thermopile located between the individual and a thermostat. Body heat power is calculated from the signals measured by the thermopile and the amount of power dissipated across the thermostat in order to maintain a constant temperature. The first prototype we built had a detection area measuring 6 × 6 cm^2^, while the second prototype, which is described herein, had a 2 × 2 cm^2^ detection area. This new design offers three advantages over the initial one: (1) greater resolution and three times greater thermal sensitivity; (2) a twice as fast response; and (3) it can take measurements from smaller areas of the body. The sensor has a 5 mW resolution, but the uncertainty is greater, up to 15 mW, due to the measurement and calculation procedure. The order of magnitude of measurements made in healthy subjects ranged from 60 to 300 mW at a thermostat temperature of 28 °C and an ambient room temperature of 21 °C. The values measured by the sensor depend on the ambient temperature and the thermostat’s temperature, while the power dissipated depends on the individual’s metabolism and any physical and/or emotional activity.

## 1. Introduction

The main developments in calorimetry instrumentation have occurred in the area of thermal analysis, a field that has observed the construction of a myriad of devices that are only differentiated by the specific characteristics of the thermal process being studied [1,2]. In calorimetry, in order to measure the heat energy produced during a process, it must take place in an enclosed, controlled area where the external factors influencing the thermodynamic process can be minimised. This work presents a calorimetric sensor designed for localised measurement of surface heat dissipated by the human body. We therefore attempt to measure the power transmitted by conduction using a thermopile placed between the human body and a thermostat maintained at a constant temperature. In this particular calorimetry application, the dissipation being measured does not occur inside the measuring device but rather outside it; this instrument does not, therefore, comply with normal calorimetry standards. The working principle for this small instrument is based on the law of conductive heat transfer and, as its operation requires a thermostat to reach a constant temperature, it can be classed as an isothermal heat conduction calorimeter [3].

The instrument has a wide variety of applications as it can be used to study different energetic processes occurring in the human body in terms of power and energy. One potential application is to control the power dissipated while using different methods to burn malignant cells. It also offers the opportunity to study dissipation from different areas of the body, to identify abnormal dissipation, or to examine body heat dissipation according to metabolism, and physical and/or emotional activity. We have already presented our first prototype with a 6 × 6 cm^2^ detection area in previous studies [4,5,6,7]. In this work, we present a second, smaller prototype with a detection area measuring just 2 × 2 cm^2^. The advantages of this second prototype are: (1) greater resolution; (2) quicker response; and (3) it can take measurements from smaller areas of the body.

Body heat measurements are currently taken for the entire body and primarily through indirect calorimetry techniques based on CO_2_ production (VCO_2_) or O_2_ consumption (VO_2_) measurements. These measurements and some empirical formulae are used to calculate the total heat dissipated by the subject in function of the physical activity being performed [8,9]. However, the instrument presented in this work provides the means for a “direct calorimetry” technique. While the modern instruments for CO_2_ and O_2_ measurement used in indirect calorimetry are actually very well developed, direct calorimetry applied to living things has seen very little development because the organism being studied must be located within an enclosed and controlled chamber [10,11]. The sensor presented here can take direct measurements and in any environment.

We should mention that a thermopile acting as a heat flow meter has been used to measure solar energy incident on a free surface; however, pyrometers are more efficient for these applications [12]. Thermopile-based systems have also been created for use in other applications designed to harness electrical energy from body heat emissions [13,14].

In previous publications [4,5,6,7], we described different aspects of the first prototype’s performance. With the present study we aim to provide a comprehensive account of the second prototype’s performance, emphasising the most important characteristics and publishing some of the experimental data obtained from measurements on the human body. We begin with a detailed description of the experimental setup, then we model the device and finally present the experimental verification of its validity. Modelling consists in treating the instrument as a linear time invariant system with two inputs, two outputs and four transfer functions. This model can be used to simulate the performance, define the sensor’s operating range and establish a method to measure the power dissipated by the human body.

## 2. Experimental Section

### 2.1. Sensor and Measurement and Control Instrumentation

**Figure 1:**
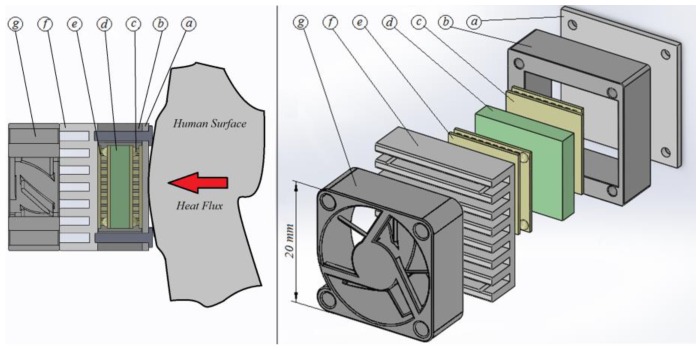
Cross-sectional diagram and exploded view of the calorimetry sensor: **a** (aluminium plate), **b** (thermal insulation), **c** (measurement thermopile), **d** (thermostat), **e** (cooling thermopile), **f** (aluminium heat sink) and **g** (fan).

The calorimetry sensor was primarily based around a 13.2 × 13.2 × 2.2 mm^3^ HOT20-65-F2A Laird thermopile (Part c in Figure 1) placed between a thermostat and a 20 × 20 × 1 mm^3^ aluminium plate (Part a in Figure 1), which was attached to the area where the heat flow is measured. The thermopile produced a calorimetric signal thanks to the Seebeck effect.

The thermostat comprised a small (10 × 12 × 3 mm^3^) aluminium block (Part d in Figure 1) containing a heating resistor (R = 16.74 Ω) and an RTD sensor (1PT100KN1515, Omega, Manchester, UK). The thermostat also included a cooling system (Parts e, f and g in Figure 1) based around a thermopile (HOT20-65-F2A, Laird, London, UK, 13.2 × 13.2 × 2.2 mm^3^), which absorbed heat from the thermostat through the Peltier effect, and an aluminium heat sink (with its corresponding fan) attached to the cooling thermopile’s hot surface.

**Figure 2:**
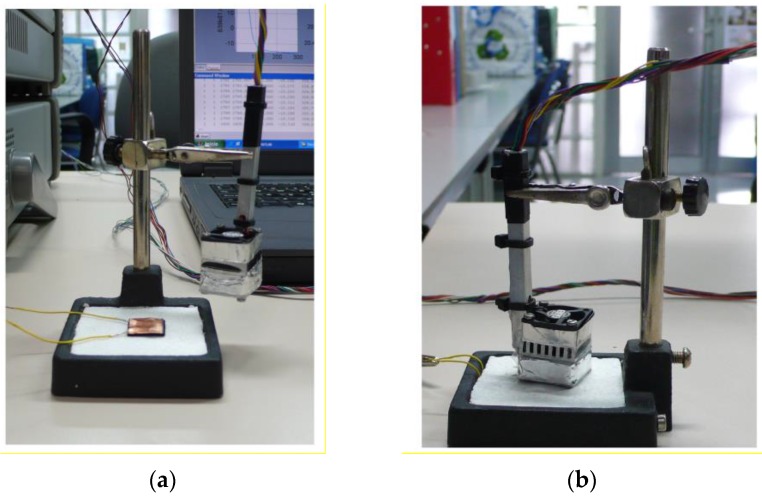
Base with the calibration resistor (**a**) and the sensor placed on the calibration base (**b**).

The sides of the entire device were thermally insulated by covering them with expanded polystyrene and reflective aluminium foil to minimise conductive and radiative heat transfer (Part b in Figure 1). The device was also equipped with a second RTD, positioned externally to measure the room temperature associated with each experimental measurement. Figure 1 shows a diagram of the sensor and its constituents: a (aluminium plate), b (thermal insulation), c (measurement thermopile), d (thermostat), e (cooling thermopile), f (aluminium heat sink) and g (fan). The device included a resistor (R = 27 Ω) contained within a 10 × 10 mm^2^ copper plate which was placed on the calibration base (Figure 2).

**Figure 3:**
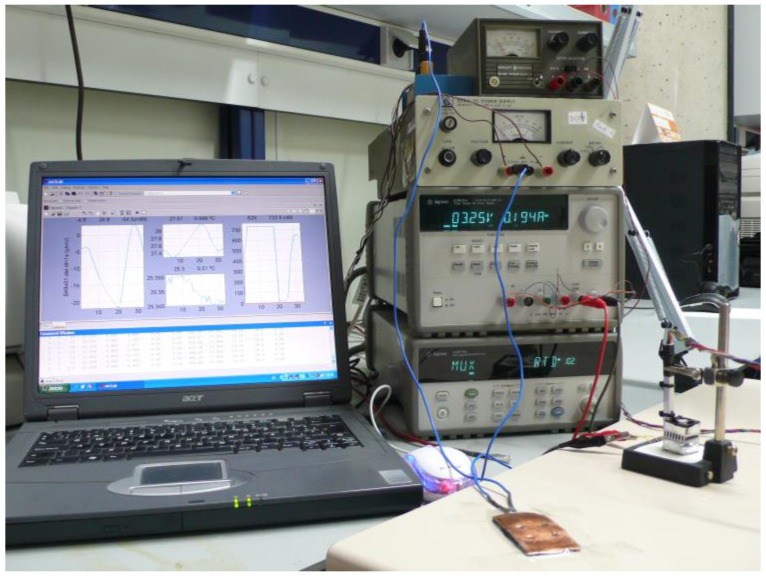
Measurement and control instrumentation.

A programmable power supply (Agilent E3631A, 80 W Triple Output Power Supply, 6 V, 5 A and ±25 V, 1 A, Keysight, Santa Clara, CA, USA) was used to power the Joule calibration resistor, the heating resistor located inside the thermostat and the Peltier cooling module. The fan was powered by an auxiliary power supply. The calorimetric signal, thermostat temperature and ambient temperature were all recorded using a data acquisition system (Agilent 34970A Data Acquisition/Switch Unit and an Agilent 34901A 20-Channel Multiplexer). We used an Agilent 82357B USB/GPIB Interface to provide a direct connection between laptop USB ports and GPIB instruments. The experimental setup was controlled with a program in MatLab^®^; the program used a PID controller to ensure the thermostat maintained a constant temperature and stored all the variables (calorimetric signal, temperatures and the power dissipated by each resistor) for subsequent data analysis. Baseline and data collection sampling periods were both set to one second. Figure 3 shows the instruments forming the measurement and control system.

The experimental setup also included a third power supply to run another resistor placed on the laboratory bench. This additional resistor was located underneath a copper plate with a larger surface area (60 × 35 mm^2^) than the sensor (20 × 20 mm^2^) and was used to simulate a measurement made on the surface of the human body (see Figure 3).

### 2.2. Sensor Operating Diagram

**Figure 4:**
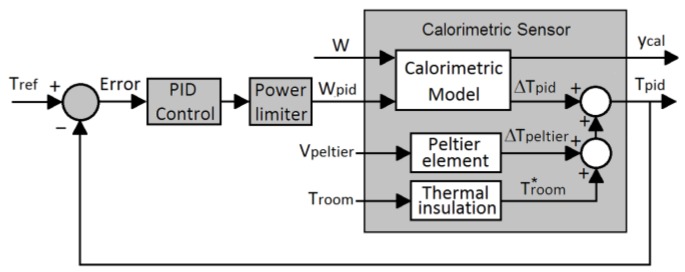
Calorimetry sensor operating diagram.

To explain how the sensor works, Figure 4 is a block diagram where each block represents a different part of the device and features its corresponding input and output variables. The diagram includes the temperature control loop for the thermostat, where *T_ref_* is the desired thermostat temperature and *T_pid_* is the thermostat’s actual temperature. The PID controller was used to calculate the power *W_pid_* dissipated in the thermostat according to the following equation:

(1) $$W_{pid}=K_{p}e(t)+K_{i}\int_{0}^{t}e(t)dt+K_{d}\frac{de(t)}{dt}$$

where the error *e*(*t*) = (*T_ref_ − T_pid_)*. Having determined the power to be dissipated, a power limiter was introduced so that 0 < *W_pid_* < (*W_pid_*)_max_. In the present case, (*W_pid_*)_max_ = 734 mW. Parameters for the PID controller were determined experimentally using the Ziegler-Nichols tuning rules [15,16] and were as follows: *K_p_* = 0.8 W·K^−1^; *K_i_* = 0.14 W·K^−1^·s^−1^; *K_d_* = 2.4 W·K^−1^·s. Hence we managed to reach a steady-state temperature (with oscillations of ±5 mK) in 200 s for a programmed 5 K temperature step.

The block representing the calorimetry sensor comprises three sub-blocks: The first sub-block is the thermal insulation block which served to attenuate the ambient room temperature *T_room_*. The second sub-block is the Peltier element whose function was to decrease the thermostat temperature, if necessary. Although the thermopile can be calibrated separately [17], we decided to perform calibration on the actual configuration. In this case, the functional relationship between the applied voltage (*V_Peltier_*) and the temperature difference (*ΔT_Peltier_*), with the fan turned on, is as follows:

(2) $$\Delta T_{Peltier}=T_{pid}-T_{room}^{*}=1.7-\;9.3\;V_{Peltier}+\;1.5\;V_{Peltier}^{2}$$

The third sub-block is the calorimetric model. Its inputs were the powers passing across the sensor (*W*) and dissipated in the thermostat (*W_pid_*), while the outputs were the calorimetric signals taken from the measurement thermopile (*y_cal_*) and any fluctuations recorded in the thermostat’s actual temperature (*ΔT_pid_*). Therefore, it is a multiple-input, multiple-output (MIMO) system.

Finally, and in accordance with the schematic in Figure 4, the thermostat’s actual temperature is given by:

(3) $$T_{pid}=T_{room}^{*}+\Delta T_{peltier}+\Delta T_{pid}$$

## 3. Sensor Modelling and Calibration

### 3.1. Modelling

**Figure 5:**
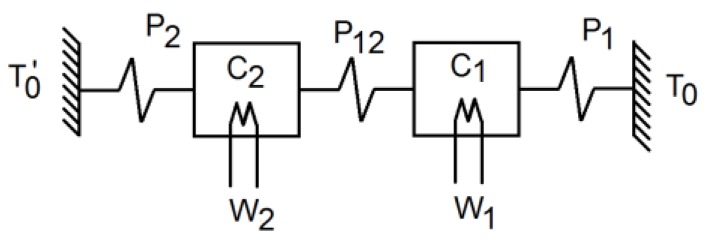
Calorimetry sensor model: *C*_1_ represents the power dissipation element and one surface of the measurement thermopile, *C*_2_ represents the thermostat and the thermopile’s other surface. *T*_0_ and *T′*_0_ represent temperatures outside the sensor. *W*_1_ and *W*_2_ are the powers dissipated in each domain.

As shown schematically in Figure 4, the model chosen for sensor identification purposes consists in treating the instrument as a linear time invariant system with two inputs, two outputs and four transfer functions. The relationship between the inputs and outputs in Laplace domain is as follows:

(4) $$\left(\begin{array}{c} \Delta Y_{cal}(s)\\ \Delta T_{pid}(s) \end{array}\right)=\left(\begin{array}{cc} TF_{1} & TF_{2}\\ TF_{3} & TF_{4} \end{array}\right)\left(\begin{array}{c} \Delta W(s)\\ \Delta W_{pid}(s) \end{array}\right)$$

where *W*(*s*), *W_pid_*(*s*), *ΔY*(*s*) and *ΔT_pid_*(*s*) are the Laplace transforms of the inputs and outputs, and each transfer function (*TF_i_*) can be calculated from:

(5) $$TF_{i}(s)=K_{i}\frac{\left(1+s\tau_{i}^{*}\right)}{\left(1+s\tau_{1})(1+s\tau_{2}\right)}$$

where *K_i_* is the sensitivity or steady-state response to a unit step, and *τ_i_ = −*1/*s_i_* and τ_i_* *=* −1/s_i_* (*s_i_* represents the poles and s_i_* the zeros for each *TF_i_*). This is equivalent to a total of four parameters for each *TF_i_*; if we force all the *TF_i_* to take the same pole, this will give a total of 10 parameters. To further reduce the number of parameters, we selected a model based on what is known as the localised-constant model, a model widely used in calorimetry [17,18,19,20]. Taking into account that the calorimetric signal depends on the temperature recorded on each surface of the measurement thermopile, we can consider two separate bodies with these temperatures. Hence, the first body represents the domain encompassing the dissipation site and one of the thermopile’s surfaces, while the second body represents the domain formed by the thermostat and the other side of the thermopile. Each body was assumed to have an infinite thermal conductivity and heat capacity *C_i_*. In addition, each body was connected to the other and to the exterior using thermal couplings with a thermal conductivity of *P_ik_* (see Figure 5).

The power *W_i_* in each domain is equal to the power required to change its temperature plus the power transmitted by conduction to the neighbouring domains, where each domain’s power can be calculated from:

(6) $$\begin{array}{l} W_{1}=C_{1}\frac{dT_{1}}{dt}+P_{12}(T_{1}-T_{2})+P_{1}(T_{1}-T_{0})\\ W_{2}=C_{2}\frac{dT_{2}}{dt}+P_{12}(T_{2}-T_{1})+P_{2}(T_{2}-T_{0}^{\prime }) \end{array}$$

If we assume that *T*_0_ and *T′*_0_ are constants and correct the baselines for all the temporal variables, then we get:

(7) $$\begin{array}{l} \Delta W_{1}=C_{1}\frac{d\Delta T_{1}}{dt}+P_{12}(\Delta T_{1}-\Delta T_{2})+P_{1}\Delta T_{1}\\ \Delta W_{2}=C_{2}\frac{d\Delta T_{2}}{dt}+P_{12}(\Delta T_{2}-\Delta T_{1})+P_{2}\Delta T_{2} \end{array}$$

Performing a Laplace transform, with null initial conditions having corrected the baselines, gives:

(8) $$\left(\begin{array}{cc} C_{1}s+P_{1}+P_{12} & -P_{12}\\ -P_{12} & C_{2}s+P_{2}+P_{12} \end{array}\right)\left(\begin{array}{c} \Delta W_{1}\\ \Delta W_{2} \end{array}\right)=\left(\begin{array}{c} \Delta T_{1}\\ \Delta T_{2} \end{array}\right)$$

Considering that the calorimetric output is proportional to the difference in temperatures:

(9) $$\Delta Y_{cal}=k(T_{1}-T_{2})=k(\Delta T_{1}-\Delta T_{2})$$

If we proceed with *W = W*_1_, *W_pid_ = W*_2_ and *ΔT_pid_ = ΔT*_2_, then we obtain the relationship between the inputs and outputs given in Equation (4), where each *TF_i_* is given by:

(10) $$\begin{array}{l} TF_{1}(s)=\frac{k(C_{2}s+P_{2})}{\Delta }\\ TF_{2}(s)=-\frac{k(C_{1}s+P_{1})}{\Delta }\\ TF_{3}(s)=\frac{P_{12}}{\Delta }\\ TF_{4}(s)=\frac{C_{1}s+P_{1}+P_{12}}{\Delta }\\ where\;\Delta =(C_{1}s+P_{1}+P_{12})(C_{2}s+P_{2}+P_{12})-P_{12}^{2} \end{array}$$

The four *TF_i_* have the same poles, but different sensitivities and zeros. With this approach we managed to reduce the number of model parameters to just six: *C*_1_, *C*_2_, *P*_1_, *P*_12_, *P*_2_ and *k*. The sensitivities, poles and zeros for each *TF_i_* were calculated from these parameters using Equation (10).

### 3.2. Calibration

The model parameters (Table 1) were determined by minimising a certain error criteria between the experimental ($\Delta y_{cal},\;\Delta T_{pid}$) and theoretical curves ($\Delta {\hat{y}}_{cal},\;\Delta {\hat{T}}_{pid}$) calculated with equations from the model (Equation (7)). We achieved this end using the Nelder–Mead simplex search algorithm [21] and MatLab software [22]. The error criterion selected (*σ*) was the mean squared error given by the equations:

(11) $$\begin{array}{l} \sigma_{y}=\frac{1}{N}\sqrt{\sum_{i=1}^{N}{\left(\Delta y_{cal}[i]-\Delta {\hat{y}}_{cal}[i]\right)}^{2}}\\ \sigma_{T}=\frac{1}{N}\sqrt{\sum_{i=1}^{N}{\left(\Delta T_{pid}[i]-\Delta {\hat{T}}_{pid}[i]\right)}^{2}}\\ \sigma =\sigma_{y}+\gamma \;\sigma_{T} \end{array}$$

**Figure 6:**
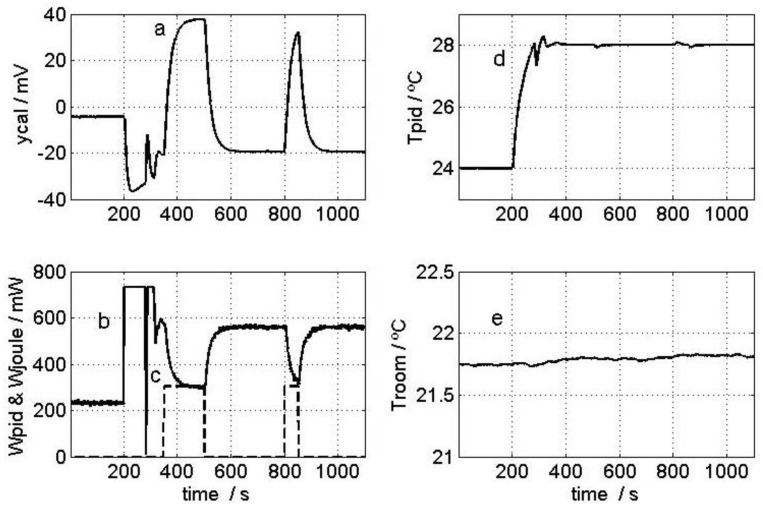
Input and output curves used in the calibration: (**a**) Calorimetric signal; (**b**) Power dissipated in the thermostat resistor; (**c**) Power dissipated into the calibration base by the Joule effect; (**d**) Thermostat temperature; and (**e**) Room temperature.

**Figure 7:**
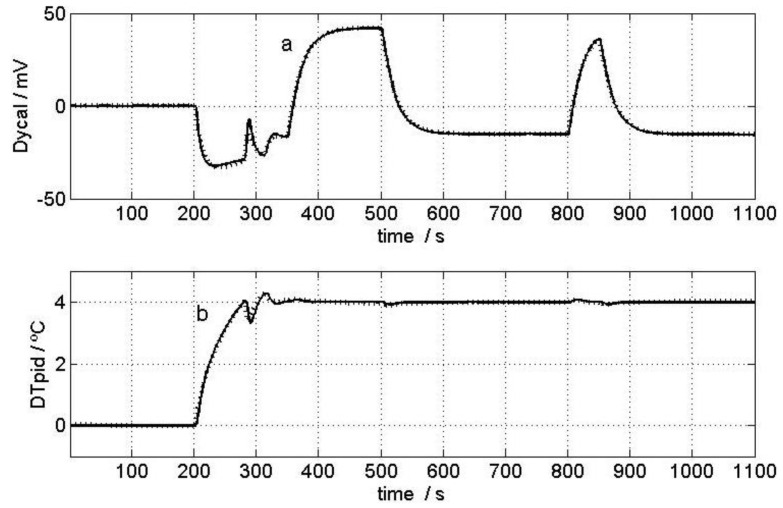
Calibration. Fit between the experimental curves (lines) and those calculated using the model (points): (**a**) Calorimetric signal; and (**b**) thermostat temperature. Curves feature a corrected baseline.

**Figure 8:**
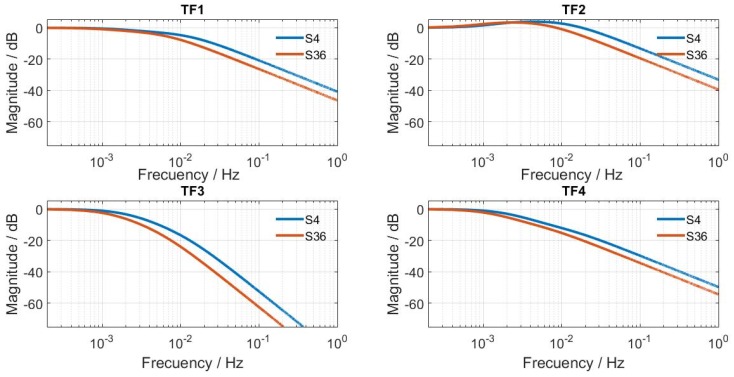
Log-magnitude curves for the transfer function given by Equations (4) and (5). S4, 4 cm^2^ minisensor described in this study. S36, an earlier 36 cm^2^ sensor (Reference [5]).

To carry out model identification, the sensor was placed on the calibration base and the thermostat set to 24 °C. Once the system reached a steady state, the thermostat was reprogrammed to 28 °C and two 117 mW pulses were simultaneously dissipated across the calibration resistor, thereby producing some significant variations in the input (*W* and *W_pid_*) and output (*y_cal_* and *T_pid_*) curves. These curves and the room temperature (*T_room_*) during measurement are shown in Figure 6. Figure 7 shows the excellent fit between the experimental output curves and those calculated with the model. Table 1 presents the result of the calibration for the minisensor described in this work (which we called S4, given the 4 cm^2^ detection area), as well as the parameters for the first prototype, S36 (with a 36 cm^2^ detection area) [5]. The static response sensitivity of the new sensor (S4) was checked and found to be higher (*K*_1_ is about three times higher). Figure 8 compares the dynamic responses of the different *TF_i_* for the S4 and S36 sensors; the magnitudes are presented in dB corresponding to each frequency, and assuming unit sensitivity (*K_i_* = 1) in all cases. *TF*_1_, *TF*_2_ and *TF*_4_ decreased by 20 dB per decade; they could then be approximated with *TF* and just one time constant, with results of 17.5, 7.3 and 48.0 s, respectively. The S36 sensor, however, had greater time constants: 33, 15 and 85 s. As expected, a smaller sensor involves lower heat capacity elements (*C_i_*) that decrease the time constants and, in our case, the response was twice as fast because the time constants of the prototype decreased by half.

## 4. Results

### 4.1. Method for Measuring and Calculating the Mean Power Dissipated from the Surface of the Human Body

**Figure 9:**
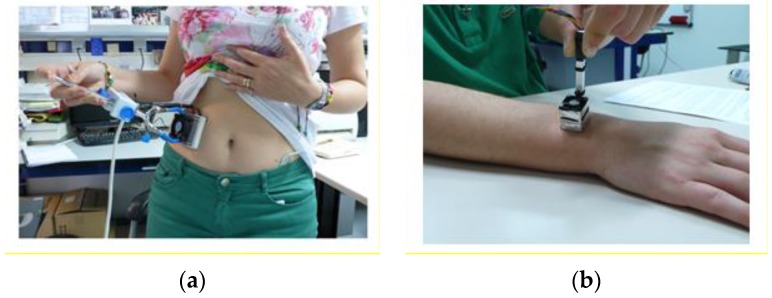
Placing the S36 (**a**) and S4 (**b**) sensors on the human body.

The instrument was operated as follows. Before taking a reading from a human body, the sensor was situated on the base until it reached the temperature set on the thermostat. The sensor was then placed on the area of the body to be measured until the thermostat again recorded a steady-state temperature (Figure 9), at which point the sensor was returned to its base. A calibration measurement by the Joule effect can be taken before and/or after taking a measurement on the human body, i.e., when the sensor is on its base.

According to the equations from the model (Equation (10)), the sensitivities (*K_i_ = TF_i_*(0)) are independent of *C*_1_ and the calibration base. This is confirmed experimentally by changing the size of the calibration resistor and observing that, while the time constants vary when this resistor is changed, the *K_i_* sensitivities remain constant. Thus, Equation (4) for a steady-state condition is transformed into an equation for the sensor that is independent of the size of surface measured:

(12) $$\left(\begin{array}{c} \Delta Y_{cal}\\ \Delta T_{pid} \end{array}\right)=\left(\begin{array}{cc} K_{1} & K_{2}\\ K_{3} & K_{4} \end{array}\right)\left(\begin{array}{c} \Delta W\\ \Delta W_{pid} \end{array}\right)$$

The calculation of the mean power *W_mean_* flowing across the sensor during the sensor application period *t_d_* consists of determining the areas under the calorimetric curves and the curves for the power developed in the thermostat after correcting the baselines:

(13) $$W_{mean}=\frac{1}{t_{d}}\int W(t)dt=\frac{1}{t_{d}K_{1}}\left(\int y_{cal}(t)dt-K_{2}\int W_{pid}(t)dt\right)$$

**Figure 10:**
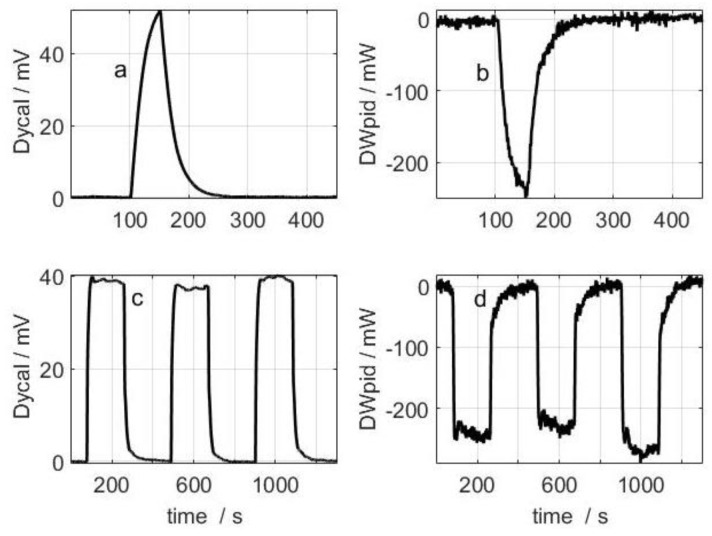
Measurement performed on the calibration base: (**a**) calorimetric signal; and (**b**) power dissipated in the thermostat. Measurement performed on the large resistor placed on the laboratory workbench: (**c**) calorimetric signal; and (**d**) power dissipated in the thermostat. The baseline was corrected for the four curves displayed.

For the dissipation in the calibration base, as shown in Figure 10a,b, the calculated *W_mean_* = 303.4 mW, while the experimental *W_mean_* = 305.1 mW; this represents a difference of −0.6% (measurement made with *T_pid_* = 28.0 °C and *T_room_* = 21.8 °C).

To check the method’s validity we took a measurement using a large resistor placed on the laboratory workbench (Figure 3); the resistor was placed in the centre, under a thin, 20 cm^2^ sheet of copper. This resistor was used to simulate a measurement on the surface of the human body, where the dissipation occurs across the whole surface yet the measurement is made on an area of just 4 cm^2^. Figure 10c,d shows three consecutive measurements taken using the following method: the sensor was placed on the calibration base and once it reached a steady-state temperature it was placed on the large resistor; the sensor was then returned to the calibration base and the process repeated three times. The results were: 191.6, 184.0 and 187.2 mW, in other words 187.8 ± 3.8 mW. In this case the large resistor dissipated 404 mW, but it must be taken into account that the surface of this resistor was 20 cm^2^, while the detection surface was 4 cm^2^ (measurement made with *T_pid_* = 28.0 °C and *T_room_* = 21.3 °C). The measured power was not proportional to the area because the dissipation was not uniformly distributed over copper surface (it was higher in the central zone).

**Figure 11:**
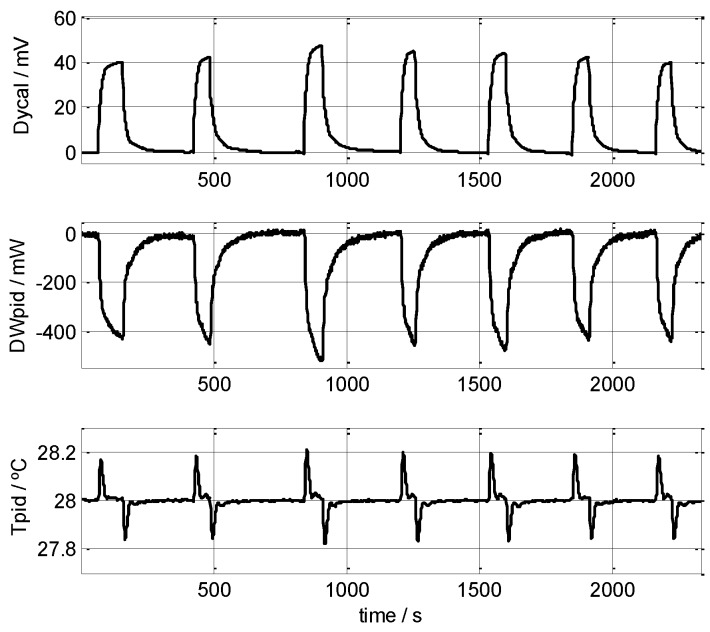
Consecutive measurements made on the left wrist of a healthy, 58-year-old male subject. Calculated mean powers: 147, 162, 173, 177, 167, 162 and 150 mW (162 ± 15 mW), at *T_room_* = 21 °C, *T_pid_* = 28 °C. The baselines of the curves for the calorimetric signal and the thermostat’s power were corrected. Thermostat temperature is also shown (*T_pid_*). *V_Peltier_* = 0.2 V.

**Figure 12:**
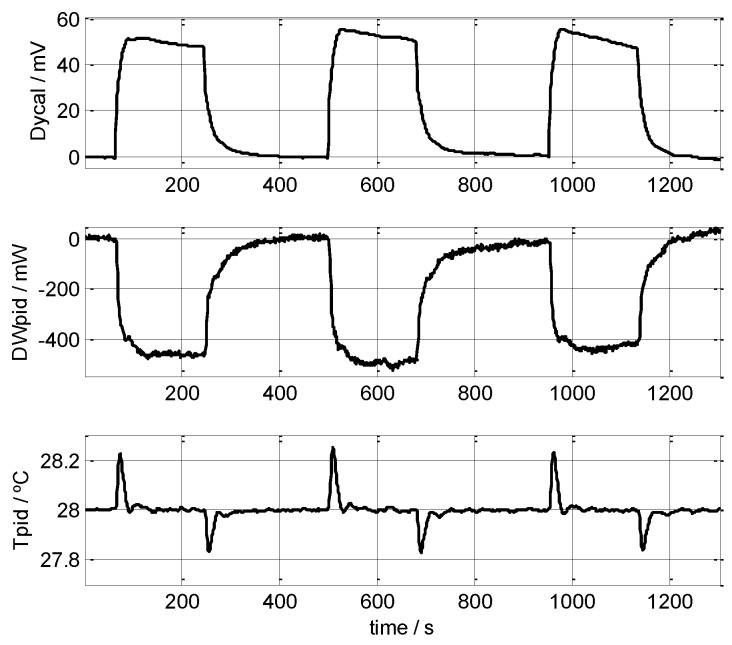
Three consecutive measurements made on the left wrist of a healthy, 22-year-old male subject. Calculated mean powers: 199, 214 and 219 mW (209 ± 10 mW) at *T_room_* = 21.4 °C, *T_pid_* = 28.0 °C. The baselines of the curves for the calorimetric signal and the thermostat’s power were corrected. Thermostat temperature is also shown (*T_pid_*). *V_Peltier_* = 0.2 V.

We performed several measurements on the human body, mainly on the left wrist. The results of this type of measurement depend on the individual’s condition; however, in this work, we did not study the measurements while correlating them against the test subject’s metabolic or emotional status, we instead focused on the reproducibility of the results recorded with the sensor. As an example, Figure 11 shows seven consecutive, short measurements (50–90 s) made on the left wrist of a healthy, 58-year-old male subject; the powers recorded were 147, 162, 173, 177, 167, 162 and 150 mW, giving a mean value of 162 ± 15 mW, measured at *T_room_* = 21.0 °C and *T_pid_* = 28.0 °C. Recordings made on the same subject but a different day gave results of 219 mW on the left wrist, 245 mW on the forehead, and 282 mW on the left pectoral region, where *T_room_* = 21.4 °C and *T_pid_* = 28.0 °C. Figure 12 displays three consecutive, long measurements (180 s) made on the left wrist of a healthy, 22-year-old male subject; the results were 199, 214 and 219 mW (209 ± 10 mW), measured at *T_room_* = 21.4 °C and *T_pid_* = 28.0 °C. Note that this last measurement took a total time of 30 minutes including the initial thermostat temperature stabilisation period. We conclude that the device and measurement method are valid, and the results obtained lie within an uncertainty of ±15 mW.

**Figure 13:**
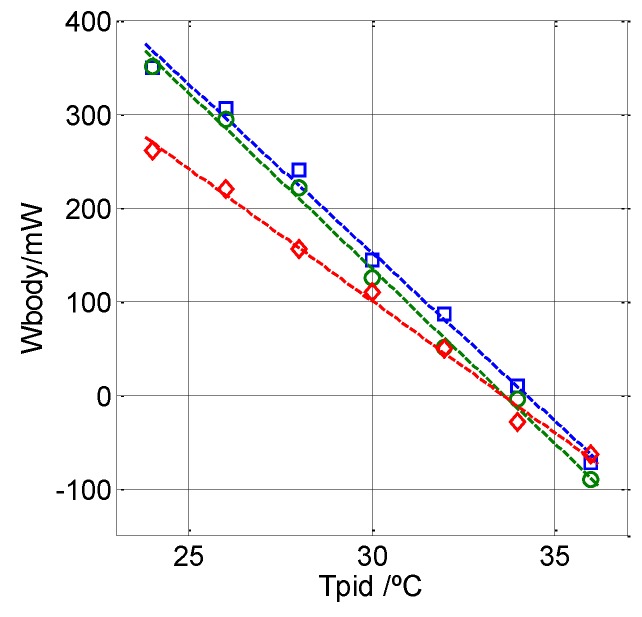
Three measurements made on the left wrist of a healthy, 58-year-old male subject on three different days (squares, circles and diamonds) for different thermostat temperatures *T_pid_* (*T_room_* = 26 °C).

We also included in this work measurements of the left wrist of a healthy 58-year-old male at different thermostat temperatures ranging from 24 °C to 36 °C and at an ambient temperature of 26 °C. Figure 13 shows three sets of measurements made on three different days. Each series consists of seven consecutive measurements, one for each thermostat temperature. The values obtained can be linearly adjusted, the place the slope cuts the horizontal axis depends on the physical and/or emotional state of the subject.

### 4.2. Sensor Resolution and Operating Domain

Operating domain refers to the thermostat’s viable temperature range (*T_pid_*) and to the minimum and maximum measurable powers (*W*). The temperature range depends on the ambient room temperature (*T_room_*). It can be calculated using Equations (3) and (12), arriving at the following equation:

(14) $$T_{pid}=T_{room}+\Delta T_{Peltier}+K_{3}W+K_{4}W_{pid}$$

The temperature decrease caused by the Peltier element is given by Equation (2). The sensitivities are *K*_3_ = 10.1 K/W and *K*_4_ = 12.0 K/W (Table 1). Initially, we shall make the following assumptions: there is no dissipation (*W* = 0), (*ΔT_Peltier_*)_min_ = −12.7 K, (*ΔT_Peltier_*)_max_ = 1.7 K, and (*W_pid_*)_max_ = 734 mW. Then, we shall calculate the minimum and maximum possible values for the thermostat temperature: (*T_pid_*)_min_ = *T_room_* − 12.7; (*T_pid_*)_max_ = *T_room_* + 10.5. However, the permissible temperature range depends on the maximum measurable power as described below.

With respect to the sensor’s operating range, we must also consider the minimum and maximum measurable powers. The minimum power is related to the noise from the signals. At steady state, the oscillations in the calorimetric signal and in the power dissipated by the thermostat are ±0.2 mV and ±10 mW, respectively; hence, the oscillations in the calculated power will be ±4.3 mW. Therefore, we can conclude that the sensor has a resolution of 5 mW. However, the measurements have a greater uncertainty because the sensor is applied to the skin manually; this translates into a varying degree of contact in function of the pressure exerted by the person taking the reading. The measurement shown in Figure 11 reveals the experimental uncertainty was ±15 mW.

The maximum measurable power is related to the possible saturation of temperature control. The sensor becomes saturated when it can no longer maintain the preset temperature, either because there is insufficient power in the thermostat (upper saturation) or the measured power is too high (lower saturation). Temperature control ensures the power dissipated in the thermostat *W_pid_* decreases, in order to maintain a constant thermostat temperature, when the input power *W* increases. Equation (14) can be used to determine a feasible value for *ΔT_Peltier_* (between −12.7 and 1.7 K) and *T_pid_* (between 26 and 30 °C) over a room temperature range of 20–30 °C; under these conditions powers of up to 855 mW can be measured without saturating the sensor. Table 2 shows the voltages that must be applied to the cooling thermopile to produce the *ΔT_Peltier_* necessary to reach the preset thermostat temperature at a given ambient temperature; in the examples presented in Table 2, the maximum thermostat power *(W_pid_)*_max_ was taken as 720 mW. In practice, the measurement program calculates and applies the voltage *V_Peltier_* based on the room temperature and the temperatures set for the thermostat.

## 5. Conclusions

We have developed a calorimetric sensor that can perform a localised measurement of surface heat dissipated from the human body. The prototype was designed to measure a square surface area covering 4 cm^2^. The model derived from the conductive heat transfer equations is valid and reliably reproduces and simulates the sensor’s operation. It therefore establishes the operating domain and a method for calculating the mean power passing over the sensor. Heat power is hard to measure, as the thermal process being studied must be insulated to minimise the effect of all external influences; in calorimetry, any measurement system operating with an uncertainty of less than 2% can be considered a success. In the present study, the working conditions were very unfavourable as the sensor was used to take measurements in an open environment, hence thermal results with a 10% uncertainty are acceptable. The sensor provides true values for surface heat dissipated from localised areas of the human body; it can measure powers of 5–855 mW for thermostat temperatures of 26–30 °C and over a wide range of room temperatures (20–30 °C). It is easy to operate and has a very quick response. It takes a total of 30 min to perform the measurement in triplicate. In conclusion, the sensor is ready for use in clinical trials.

## Figures and Tables

**Table 1:** Calibration results. RC model (Equation (7)) and *TF_i_* (Equation (5)) parameters. S4, the minisensor described in this study; S36, an earlier 36 cm^2^ model (Reference [5]).

RC Model		TF Model		
S4 Sensor			S4 Sensor	S36 Sensor
*C*_1_	2.9146 J·K^−1^	*K*_1_	148.7 mV·W^−1^	46.0 mV·W^−1^
*C*_2_	3.9735 J·K^−1^	*K*_2_	−45.4 mV·W^−1^	−9.3 mV·W^−1^
*P*_1_	0.0203 W·K^−1^	*K*_3_	10.1 K·W^−1^	1.76 K·W^−1^
*P*_2_	0.0663 W·K^−1^	*K*_4_	12.0 K·W^−1^	1.70 K·W^−1^
*P*_12_	0.1116 W·K^−1^	*τ*_1_	82 s	147 s
*K*	24.6678 mV·K^−1^	*τ*_2_	13 s	27 s
Errors (Equation (11))		*τ*_1_*	60 s	91 s
*σ_y_*	29.5 µV	*τ*_2_*	144 s	243 s
*σ_T_*	1.5 mK	*τ*_3_*	0 s	0 s
*N*	1100 points	*τ*_4_*	22 s	49 s

**Table 2:** Sensor operating domain for a thermostat temperature (*T_pid_*) range of 26–30 °C and at an ambient temperature (*T_room_*) of 20–30 °C. The table shows the voltage *V_Peltier_* across the cooling thermopile required to produce the temperature decrease *ΔT_Peltier_*, and the maximum measurable power (*W*_max_) for each example.

*T_pid_*	*T_room_*	*V_Peltier_* (V)	*ΔT_Peltier_* (°C)	*W*_max_ (mW)
26 °C	20 °C	0.51	−2.64	855
	25 °C	1.26	−7.64	855
	30 °C	2.88	−12.64	855
28 °C	20 °C	0.26	−0.64	855
	25 °C	0.93	−5.64	855
	30 °C	1.92	−10.64	855
30 °C	20 °C	0.04	+1.36	855
	25 °C	0.64	−3.64	855
	30 °C	1.45	−8.64	855
